# Chimpanzee drumming: a spontaneous performance with characteristics of human musical drumming

**DOI:** 10.1038/srep11320

**Published:** 2015-06-17

**Authors:** Valérie Dufour, Nicolas Poulin, Charlotte Curé, Elisabeth H. M. Sterck

**Affiliations:** 1 Institut Pluridisciplinaire Hubert Curien, University of Strasbourg,23 rue Becquerel 67087 Strasbourg, France; 2 Centre National de la Recherche Scientifique, UMR 7178, 67087 Strasbourg, France; 3 Biomedical Primate Research Center, Ethology Research, Lange Kleiweg 161, 2288 GJ Rijswijk, the Netherlands; 4 CeStatS, Institut de Recherche Mathématique Avancée, UMR 7501, Université de Strasbourg & CNRS, 7 rue René-Descartes, 67000 Strasbourg, France; 5 CEREMA - DTer Est, Acoustics Group, F-67035 Strasbourg Cedex 2, France; 6 Utrecht University, Animal Ecology, Padualaan 8, 35845 CH, Utrecht, the Netherlands

## Abstract

Despite the quintessential role that music plays in human societies by enabling us to release and share emotions with others, traces of its evolutionary origins in other species remain scarce. Drumming like humans whilst producing music is practically unheard of in our most closely related species, the great apes. Although beating on tree roots and body parts does occur in these species, it has, musically speaking, little in common with human drumming. Researchers suggest that for manual beating in great apes to be compared to human drumming, it should at least be structurally even, a necessary quality to elicit entrainment (beat induction in others). Here we report an episode of spontaneous drumming by a captive chimpanzee that approaches the structural and contextual characteristics usually found in musical drumming. This drumming differs from most beating episodes reported in this species by its unusual duration, the lack of any obvious context, and rhythmical properties that include long-lasting and dynamically changing rhythms, but also evenness and leisureliness. This performance is probably the first evidence that our capacity to drum is shared with our closest relatives.

Producing music, performing, singing and dancing along with others is a universal part of human behaviour^1^. Scientists generally agree that several features are common to most human musical production^2^, including intentionality, decontextualisation, formality (evenness, isochrony) and joint coordination. To address the evolutionary origins of our musical skills, anthropologists, biologists and musicologists have examined the similarities between human performances and sound production in non-human animals^3^, and most agree that several features of our musical abilities are found in other species^4^,^5^,^6^. Bird or whale songs are considered analogous to music, because like many human songs they are complex vocalizations with a potential for cultural evolution, i.e they can be learned from others^7^,^8^. In great apes, manual beating is considered homologous to human drumming, because this shared capacity may reflect a common ancestral predisposition to produce music^4^.

However, manual beating by great apes generally lacks an essential characteristic of human drumming, namely evenness^9^. Isochrony, the fact of beating at regularly spaced time intervals makes the occurrence of the next beat(s) predictable, and gives a formal quality to the performance. The evenness together with leisureliness facilitates temporal coordination from others, and more generally entrainment. This remarkable feature of human music is far from being widespread in our closest relatives. Beating on tree buttresses, cans, body parts or objects, as wild chimpanzees, bonobos and gorillas do^10^,^11^,^12^,^13^,^14^,^15^, may be the sign of a link between body movement and vocal production^1^ and is sometimes called “drumming”^4^,^10^. In essence, however, it is more a spectacular noise-making display than a structurally isochronous performance^9^. These behaviors therefore have little in common with the structural, and contextual characteristics found in the musical human drumming^9^. Here, we report an unusual performance of a chimpanzee named Barney. Barney was observed beating repeatedly and spontaneously on an upturned bucket for several sequences within a period of few minutes (Supplementary Audio file and Fig. 1). We evaluated his performance to establish which features fit with the type of characteristics generally associated with human drumming^2^, i.e. intentionality, decontextualisation and formality, and explored if and how this particular event differed from previously reported manual beating displays by apes.

## Results

### Intentionality

**Figure 1:**
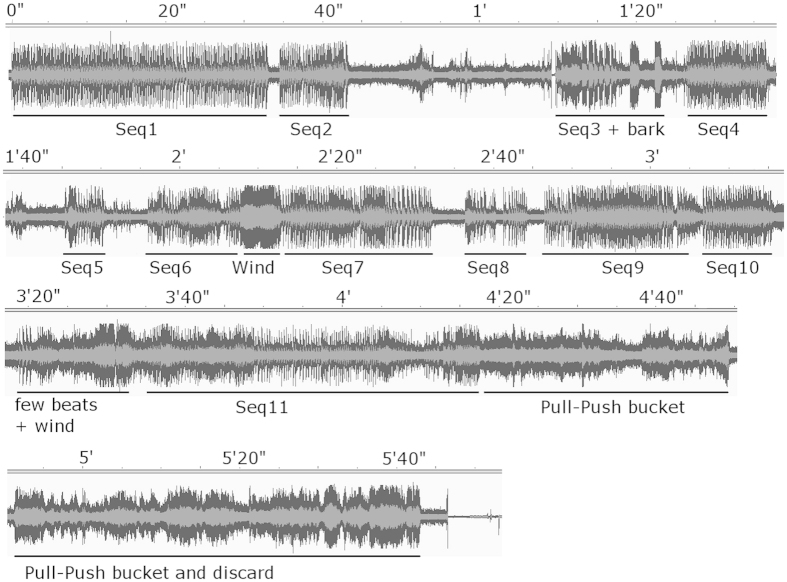
Illustration of the total drumming bout and its division into 11 sequences. Sequence 5 has less than 20 inter-beat durations and was not analyzed. At the end of the bout, the chimpanzee is on his arms and legs, quietly pushing and pulling the barrel on the floor in slow and wide circular movements. This part was not analyzed. Moving averages of the inter-beat duration of each sequence can be found in Supplementary Figure S1.

**Figure 2:**
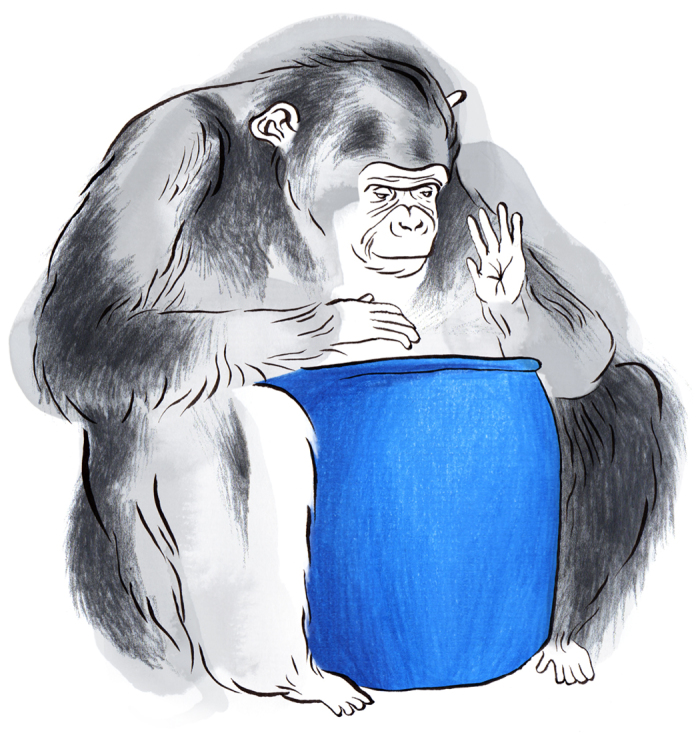
Illustration of Barney’s position when drumming manually on the barrel. The facial expression was neither tense nor playful, and the feet (and sometimes the mouth) were used to firmly hold the barrel. Illustration by *Camille Martin (School of Decorative Arts, Strasbourg).*

Assessing intentionality in non-verbalizing beings is a complex task. While means can be found to investigate it within non-human social interactions^16^, it is more difficult to pinpoint in solitary acts without an objective method of investigation. Yet in this particular drumming, Barney produced more than 685 drumbeats spread over 11 sequences for over four minutes (Fig. 1, and Supplementary Fig. S1). He remained in a seated position with the bucket between his feet, sometimes using his mouth to keep it in place (Fig. 2). The focus he showed during beating indicates that it was more than just a short, uncontrolled noise-making display. In this respect, it differs from most of the drumming episodes described in chimpanzees, who generally produce intense beating bouts of only few seconds^12^.

### Decontextualization

Human drumming does not necessarily have a particular purpose or context. This decontextualisation is not seen in great apes, whose manual beating is generally associated with play^15^, aggressive display, sexual arousal^12^ or travel^11^. In contrast, Barney had isolated himself from the others in his outside enclosure. He did not show any facial expression or postures typical of play (play face, excitement), nor did he move or exhibit aggressive display (body hair was not raised). One short but remarkable sequence (sequence 3) exhibited a change in the beating pattern and was punctuated by a bark, which could indicate a display context, but this explanation is not likely as no pilo-erection was observed. Barney did not move from his seated position, and resumed drumming after a few seconds of silence for another long sequence. This situation supports the notion that the performance was indeed decontextualized.

### Formality

**Figure 3:**
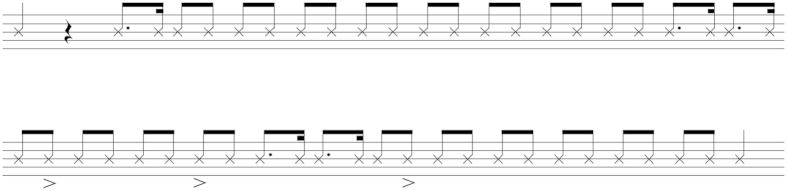
Musical translation of sequence 4. This illustrates the binary rhythm (from 1’25” in Supplementary Audio S1).

In measured music, drumming has a formal quality^2^ where sounds are produced in organized patterns such as periodicity or rhythm. Isochrony of the inter-beat intervals is one way to achieve evenness, a necessary quality to allow entrainment. In the drumming we recorded, 10 of the 11 recorded sequences were long enough to be analyzed for their rhythmical properties, with an average inter-beat duration of 245 ms (Table 1). Recorded sequences were considered as time series in which lag denotes the range between the equally spaced instants of measurement. Here our instants of measurement are the beats and the measure is the inter-beat duration. First, we performed a portmanteau test for each recorded sequence, i.e. a Ljung-Box test, to detect potential non-random patterns in the time series. Five sequences showed non-random patterns (Supplementary Fig. S2). In four of them, these patterns were still detectable over at least 15 consecutive lags, indicating a long-lasting dependency over the course of the sequence (for sequences 1, 4, 9 and 11). In the last sequence (sequence 6), patterns occurred over the next lag, thus changing more frequently. We then ran an autocorrelation procedure for the sequences where non-random patterns were detected, in order to test the linear dependency between each consecutive inter-beat duration (Supplementary Fig. S3). Dependency up to lag 3, for example, indicates that whatever the position in the sequence, the duration of the next three lags can be predicted. Significant dependencies were found in the five sequences, ranging from dependency between one inter-beat duration and the next (sequence 6) to dependency between 12 consecutive inter-beat durations (sequence 4). This shows that the beating in these 5 sequences was not only regular, but was even extremely so on occasions, with an average tempo of 257 beats per minute (bpm). This pace is close to human tempo for rhythmic music^17^. Given that this pattern is probably due to a bimanual beating, it can therefore be argued that the basic tempo averages 128 bpm, a tempo typical of rhythmic popular dance music in many humans^17^. Remarkably, there is an alternation of positive and negative correlations in sequence 4, meaning that a long inter-beat duration was followed by a short inter-beat duration, or vice-versa. Given the unexpected regularity of this pattern, we provide a musical notation for this sequence, which illustrates a binary rhythm that musicians can now replicate (Fig. 3). Finally, a fluctuation analysis tested for linear trends (acceleration or deceleration) in the dynamic of each sequence. Changes in dynamics, i.e a deceleration, were observed in sequences 1, 9 and 11. Thus, the rhythm varied from one sequence to the next, which counters the possibility that the rhythm may simply result from endogenous synchronization or from a “motoric ceiling effect” in the frequency of slapping to maximize the noise output^9^. On the contrary, Barney demonstrated evenness at leisurely, spaced time intervals. This is a novel finding, as these two characteristics are not generally associated with typical manual beating by chimpanzees.

## Discussion

Patterns in periodicity or rhythm such as those described above may help other musicians or the audience to “join in” during communal music making. We have no evidence that Barney’s drumming could have elicited any joining from others, or was even designed to do so, as he performed at a distance from the rest of his group. His performance nevertheless shares many of the characteristics of human drumming. Within other animal species, joint coordination is found in sound-mimicking birds like cockatoos^18^ and parrots^19^,^20^, but also in sea lions^21^, which can be trained to bob their head in rhythm to a song. Most studies suggest that it is difficult for monkeys to even perceive a beat, let alone synchronize their movements with it^22^,^23^. The great apes may be capable of synchrony, as seen when the chimpanzee Ai spontaneously pressed two keys in time to a 600 ms inter-beat interval auditory stimulus without previous training^24^. We do know that in the wild, chimpanzees can show loose behavioral coordination when rain dancing^25^, chorusing (when the call of one aroused individual elicits joining from others) or engaging in carnival displays^26^,^27^, but we have no evidence that these behaviors could be beat-based rather than simply due to emotional contagion^28^,^29^. They cannot therefore be directly compared to the type of “on-the-beat” synchrony shown by humans when they play music^1^. In comparison, human children can synchronize to external drumming from around the age of three onwards with an accuracy that increases as they grow older^30^.

It is rare for animal sound production to simultaneously possess more than one of the previously cited characteristics found in human music^2^. In this respect, Barney’s drumming is exceptional. It was not only decontextualised and showed formality, but also appears to be intentional. Barney’s drumming is a rare example, as other reported musical drumming in great apes, although suggestive, did not address each of this features separately. Musical drumming has been reported in a language-trained bonobo called Kanzi^31^, but published data are lacking. A recent report describes how two young chimpanzees^14^ repeatedly hit a nearby clay pot, and appeared to be attracted by the sound it made, hitting it 199 times, but no analysis of its rhythmical pattern has been provided. Barney’s performance confirms that the chimpanzee, our closest relative, could indeed be capable of drumming like a human.

According to Mithen^5^, music played a crucial role in our evolution before the existence of articulated language and improved communication between humans. By conveying emotions, and thus meaning, it is thought to have further bonded human groups, improving coordination and cohesion. Some authors have suggested a similar cohesive outcome in the drumming of great apes^11^. Other researchers also suggest that synchronized beating and chants by groups of individuals would have made the performance louder and more likely to be heard by travelling females, the dispersing gender in humans and chimpanzees^1^. Although there is very little fossil evidence regarding the birth of our musical abilities, our data are probably the first strong evidence of an evolutionary link between wild beating in chimpanzees and our own musical origins.

## Methods

### Subject & procedure

At the time of our study, Barney was a captive-born, 24-year-old male belonging to a group of 5 adult male chimpanzees raised at the Biomedical Primate Research Centre in the Netherlands. Barney was a rather low-ranking male but was not particularly withdrawn from the group and had not be seen to display stereotypical behaviors. All members of this group often noisily manipulated plastic bottles, buckets and branches while performing display (VD, personal observations). Environmental noises included road traffic and construction building noises close by. The chimpanzees also sometimes had a radio playing in the building. The drumming was spontaneous (not artificially elicited or encouraged by human presence) and occurred in January 2005 in the outside part of their enclosure. The observer (VD) was nearby at the start of the drumming, and quietly approached Barney’s enclosure to record it on a voice recorder (type Sony M527V). No camera was available within reach to film this episode. The barrel used for the drumming was part of the objects available at all time in the enclosure and was neither new, nor newly introduced to the chimpanzee. We delimited sequences as follow: first, after a pause of two seconds, we considered the following beats to be the beginning of the next sequence. Second, if sound pollution occurred (caused by wind or when the noise of the beat was covered by background noise), the inaudible part of the sequence was not analyzed, and we considered the following audible part as a new sequence. For example, because the first few beats of Sequence 6 were difficult to extract from the background noise, we started the analysis at the 6^th^ beat. The recording of Sequence 6 was interrupted by the wind. The following beats were treated separately as Sequence 7. Additionally, the analysis started from beat 3 in Sequence 4 due to a discontinuity between the second and the third beats (a one second pause). To analyze rhythmical patterns, we selected sequences with more than 20 beats (thus discarding sequence 5). Note also that the recording was stopped after the end of sequence 2, as the observer thought that the bout was over. It was immediately restarted when Barney began drumming again a few seconds later, but the first few beats of sequence 3 are missing.

The analysis was conducted using Reaper software. We used the transient detection settings tool (threshold detection was set at −24 dB and sensitivity at 25%, 60% and 75%) to measure the inter-beat durations in each sequence. For each sequence, data were analyzed using R^32^. P level of significance was set at 0.05.

### Ethics

The study was conducted in accordance with the approved scientific reports guidelines. Due to the observational nature of this study (the audio recording of a spontaneous behavioral sequence initiated by the chimpanzee), this study does not classify as an experiment as assessed by the Biomedical Primate Research Centre Animal welfare officer. Thus no additional permission from the institutes animal experiment committee was required. The study was conducted in accordance with relevant Dutch laws and in agreement with international and scientific standards and guidelines.

## Additional Information

**How to cite this article**: Dufour, V. *et al.* Chimpanzee drumming: a spontaneous performance with characteristics of human musical drumming. *Sci. Rep.* **5**, 11320; doi: 10.1038/srep11320 (2015).

## Supplementary Material

Supplementary Information

Supplementary Audio S1

## Figures and Tables

**Table 1:** Descriptive data.

	Descriptive data				Ljung-Box Test			Auto-correlation for the next n beats (b)	Dynamics		
	Mean Inter-beat (Ib) Duration (in ms)	min Ib Duration (ms)	max Ib Duration (ms)	Total N° of beats	Value (a)	khi	P_value_(at 1^rst^ lag)		T value	R Square	P
**Seq1**	212.9	95	432	155	15	14.52	<0.01	2	4.17	0.09	<0.01
**Seq2**	253.9	86	489	34	na	0.09	0.76	na	−0.45	−0.02	0.66
**Seq3**	211.9	53	1156	34	na	0.12	0.72	na	0.69	−0.01	0.49
**Seq4**	222.7	130	312	47	15	16.5	<0.01	12	0.17	−0.02	0.87
**Seq6**	217.3	88	617	57	1	6.57	0.03	1	1.78	0.04	0.08
**Seq7**	267.4	79	470	71	na	0.37	0.54	na	0.1	0.0001	0.32
**Seq8**	274.6	96	1048	28	na	0.48	0.48	na	1.23	0.02	0.229
**Seq9**	258.2	66	709	71	15	9.8	<0.01	4	3.05	0.11	0.01
**Seq10**	276.3	96	421	32	na	0.002	0.96	na	−0.9	−0.01	0.37
**Seq11**	253.8	98	726	155	15	29.09	<0.01	10	2.37	0.03	0.02

Descriptive data on inter-beat (Ib) duration, number of beats, value and results of the Ljung-Box test, the auto-correlation test and the dynamic results for each sequence.

For example, in Sequence 1, the Ljung-Box test indicates a significantly non-random pattern at the first lag, checking for the next lags indicates that non-random patterns are detectable within up to 15 lags (Supplementary Fig. S2). The autocorrelation test indicates that whatever the position in the sequence, the time at which the next 2 beats will occur can be predicted (Supplementary Fig. S3). Here, the dynamic analysis shows a significantly positive linear trend, thus an increase in the duration between two beats (deceleration).

^(a)^Number of lags within which non-random patterns can be detected (Supplementary Fig. S2).

^(b)^Number of beats where dependency between two beats is statistically significant (Supplementary Fig. S3).
