# Supplementary material for: Chimpanzee drumming: a spontaneous performance with characteristics of human musical drumming
Source: Sci Rep. 2015 Jun 17;5:11320. doi: 10.1038/srep11320 (PMC4469965; doi:10.1038/srep11320)
Supplement: Supplementary Information [file srep11320-s1.pdf]

Supplementary information

Chimpanzee drumming: a spontaneous performance with characteristics of human musical drumming

Dufour, V., Poulin, N., Curé, C., Sterck, E.H.M.

Supplementary information

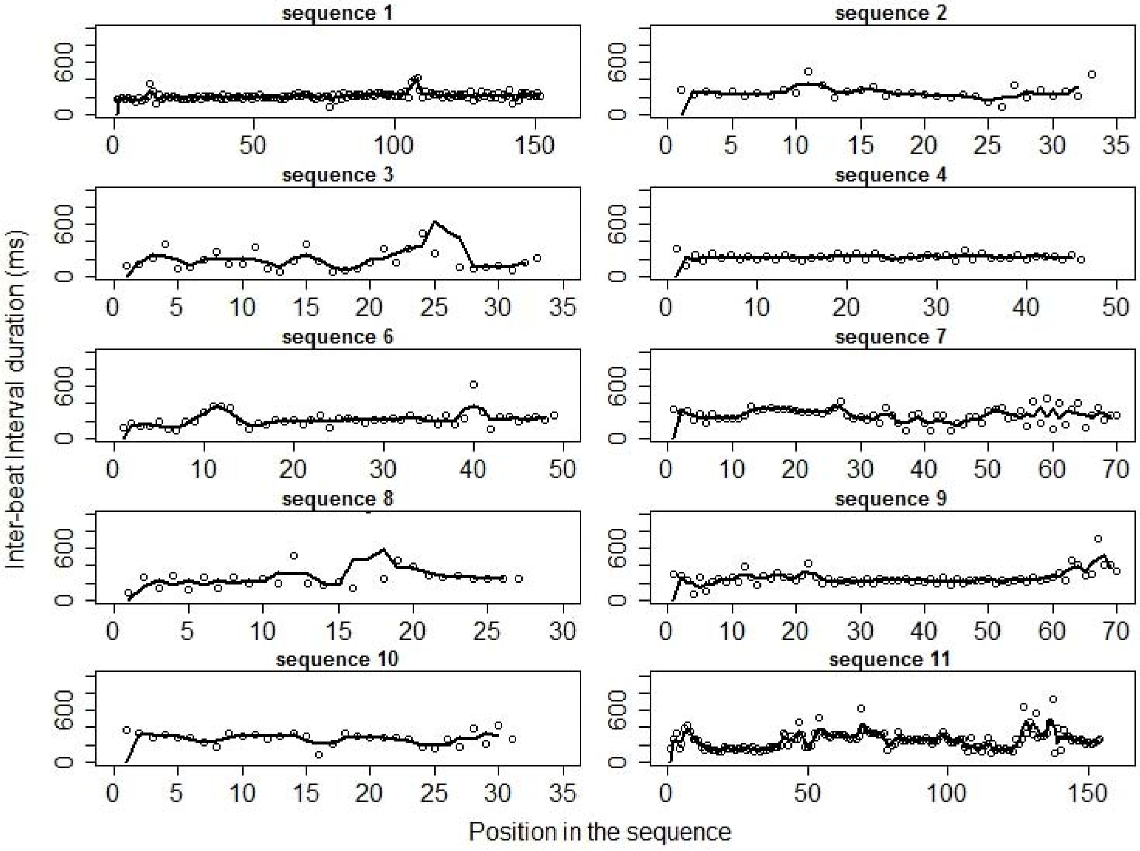

Supplementary Fig. S1: Moving average of the inter-beat durations (Y-Axis) according to their position in the sequence (X-Axis) for each sequence.

Supplementary information

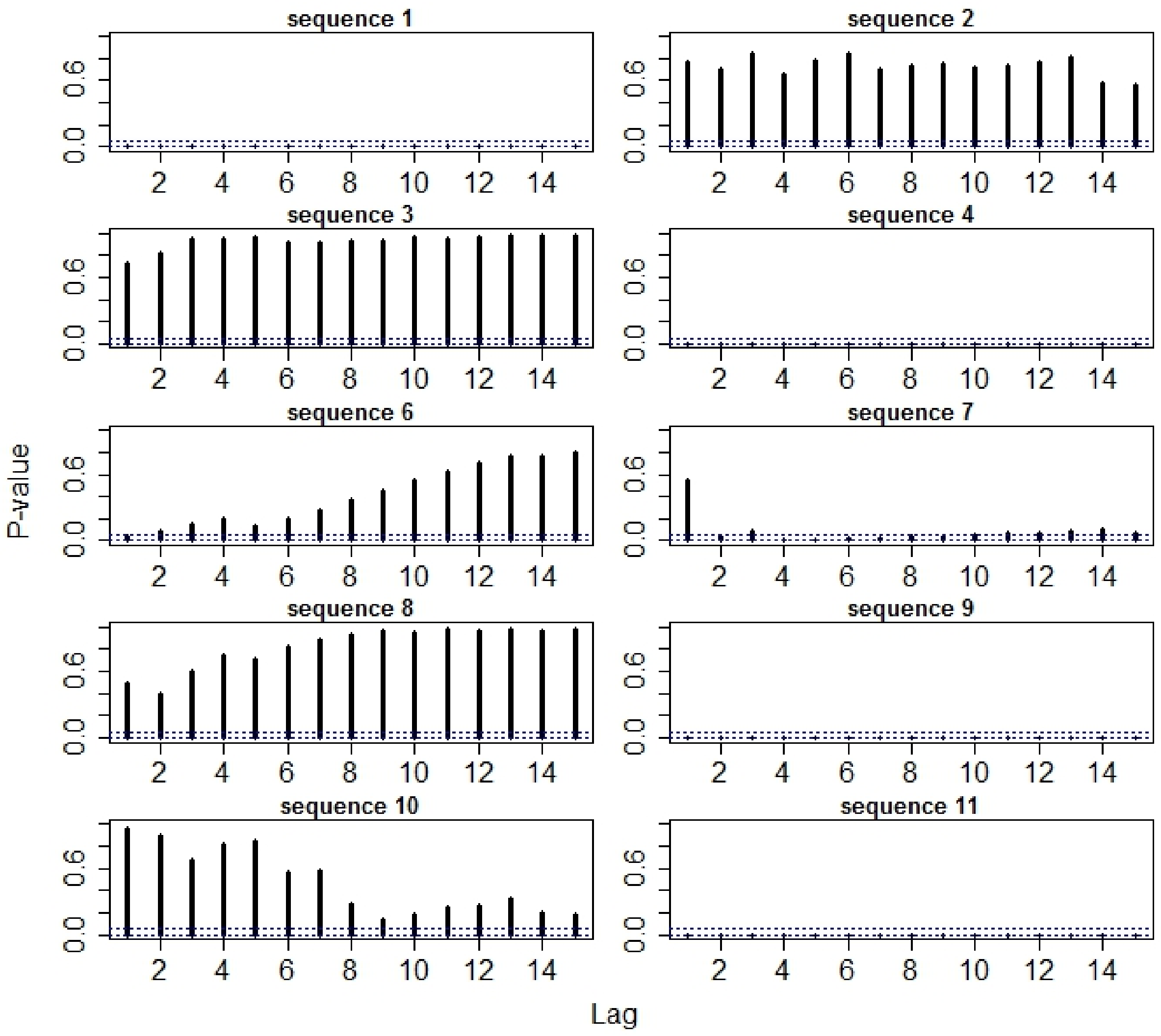

Supplementary Fig. S2: The test returns statistically significant results when the vertical line remains lower than 0.05. Used as a portmanteau test, the Ljung-Box test can provide several p-values, with one for each lag. When working with time series, lag denotes the range between the equally spaced instants of measurement. Here our instants of measurement are the beats and the measure is the inter-beat duration. Hence when considering a given lag, for example the third lag, we consider the relationship between one inter-beat duration and the one measured 3 beats later. If the analysis leads to significant p-values, this indicates that the data are not drawn at random and that there is a pattern in the time series. Patterns can be detected within up to 15 lags in sequences 1, 4, 9 and 11 and within the next lag in sequence 6.

Supplementary information

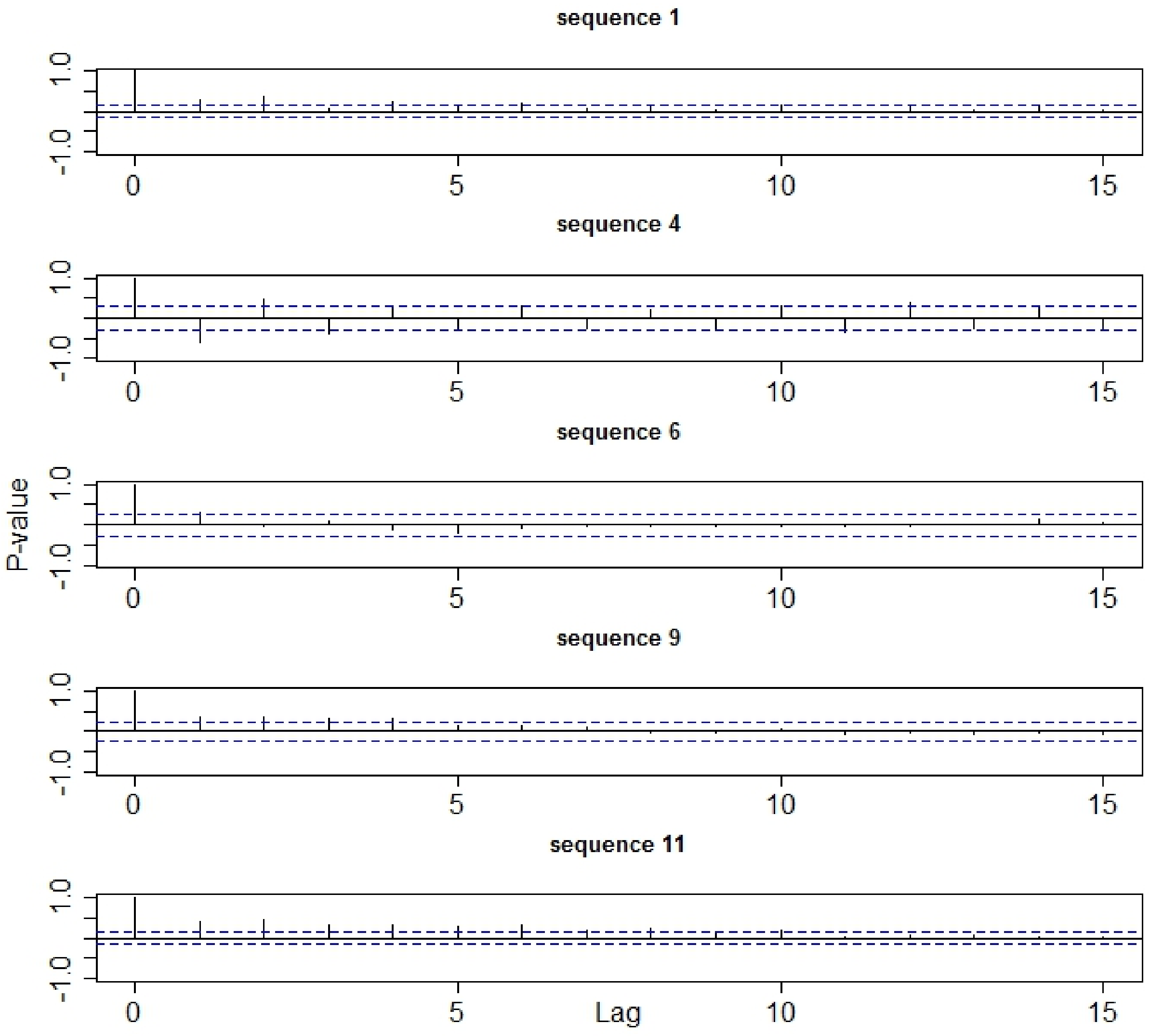

Supplementary Fig. S3: Autocorrelation test results. Autocorrelation tests results for sequences 1, 4, 6, 9 & 11 showing the level of significance (Y-axis) according to the lag considered (X-Axis). The test returns statistically significant results when the vertical line reaches or crosses the blue lines. Whatever the position in the sequence, the duration of the next inter-beat duration is significantly correlated for up to 2 lags in Sequence 1; 12 lags in sequence 4; 1 lag in Sequence 6, 4 lags in Sequence 9 and 10 lags in Sequence 11. Note that for sequence 4, the alternation of long (above the central X-axis) and short lags (below the central X-axis) is highly noticeable.
